# Effects of Size and Surface Properties of Nanodiamonds on the Immunogenicity of Plant-Based H5 Protein of A/H5N1 Virus in Mice

**DOI:** 10.3390/nano11061597

**Published:** 2021-06-17

**Authors:** Thuong Thi Ho, Van Thi Pham, Tra Thi Nguyen, Vy Thai Trinh, Tram Vi, Hsin-Hung Lin, Phuong Minh Thi Nguyen, Huyen Thi Bui, Ngoc Bich Pham, Thao Bich Thi Le, Chi Van Phan, Huan-Cheng Chang, Wesley Wei-Wen Hsiao, Ha Hoang Chu, Minh Dinh Pham

**Affiliations:** 1 Institute of Biotechnology, Vietnam Academy of Science and Technology, 18-Hoang Quoc Viet, Cau Giay, Hanoi 100000, Vietnam; hothithuong@ibt.ac.vn (T.T.H.); phamthivan1103@gmail.com (V.T.P.); nguyentrapbc@gmail.com (T.T.N.); vy.tt.h2@gmail.com (V.T.T.); vbt576@gmail.com (T.V.); minhphuongibt@gmail.com (P.M.T.N.); huyenbe82@gmail.com (H.T.B.); pbngoc@ibt.ac.vn (N.B.P.); ibtthao@gmail.com (T.B.T.L.); pvchi@yahoo.com (C.V.P.); 2 Faculty of Biotechnology, Graduate University of Science and Technology, Vietnam Academy of Science and Technology, 18-Hoang Quoc Viet, Cau Giay, Hanoi 100000, Vietnam; 3 Faculty of Medical Biotechnology—Plant Biotechnology—Pharmacology, University of Science and Technology of Hanoi (USTH), 18-Hoang Quoc Viet, Cau Giay, Hanoi 100000, Vietnam; 4 Institute of Atomic and Molecular Sciences, Academia Sinica, Taipei 10617, Taiwan; airforceone16@gmail.com (H.-H.L.); hchang@gate.sinica.edu.tw (H.-C.C.); 5 Department of Chemical Engineering, National Taiwan University of Science and Technology, Taipei 106335, Taiwan; weshsiao@mail.ntust.edu.tw

**Keywords:** nanodiamond, size effect, surface properties, H5 protein, avian influenza virus A/H5N1

## Abstract

Nanodiamond (ND) has recently emerged as a potential nanomaterial for nanovaccine development. Here, a plant-based haemagglutinin protein (H5.c2) of A/H5N1 virus was conjugated with detonation NDs (DND) of 3.7 nm in diameter (ND4), and high-pressure and high-temperature (HPHT) oxidative NDs of ~40–70 nm (ND40) and ~100–250 nm (ND100) in diameter. Our results revealed that the surface charge, but not the size of NDs, is crucial to the protein conjugation, as well as the in vitro and in vivo behaviors of H5.c2:ND conjugates. Positively charged ND4 does not effectively form stable conjugates with H5.c2, and has no impact on the immunogenicity of the protein both in vitro and in vivo. In contrast, the negatively oxidized NDs (ND40 and ND100) are excellent protein antigen carriers. When compared to free H5.c2, H5.c2:ND40, and H5.c2:ND100 conjugates are highly immunogenic with hemagglutination titers that are both 16 times higher than that of the free H5.c2 protein. Notably, H5.c2:ND40 and H5.c2:ND100 conjugates induce over 3-folds stronger production of both H5.c2-specific-IgG and neutralizing antibodies against A/H5N1 than free H5.c2 in mice. These findings support the innovative strategy of using negatively oxidized ND particles as novel antigen carriers for vaccine development, while also highlighting the importance of particle characterization before use.

## 1. Introduction

ND is a novel nanomaterial which belongs to the carbon family with excellent biocompatibility and optical properties [1]. They are very small and versatile in surface or lattice features [2]. ND has emerged as an exciting nanomaterial in recent years in many fields of research due to its unique characteristics [3,4,5,6]. Prior characterization has demonstrated NDs superior mechanical and physical characteristics, as well as its large surface area, biocompatibility, and tuneable surface structures [7]. These characteristics make ND well-suited for biomedical applications.

NDs can be produced by several methods, namely chemical vapor deposition, HPHT synthesis, and detonation [3,5]. The different production methods generated various types of ND with distinct surface properties, structure, size, and shape [8]. NDs generated by HPHT presses has broad size range (usually 30–100 nm), and are monocrystalline. These HPHT-NDs might be completely purified, and be surface oxidized by strong acid washes and air oxidation. In contrast, NDs are produced by the shock wave resulting in DNDs with narrow particle size distribution of 4–5 nm [9].

NDs can be applied in vaccinology owing to the fact that bacteria and viruses have micro- and nano-sizes [10]. They have the potential to act as an adjuvant to deliver antigens or a type of novel vaccine carrier system that can be recognized by the immune system [11]. Based on the intrinsic hydrophobicity of strong acid-oxidized NDs, their surface can conjugate with native membrane protein, as well as soluble proteins [4]. The remarkably high affinity of NDs and recombinant proteins against viruses, as well as the effect of NDs, have been demonstrated in several reports [12,13,14]. In our previous study of Pham and colleagues, the nanoconjugate containing the viral protein HA/H7N9 attached on the surface of oxidized NDs with the size in the range from ~50 nm to ~500 nm in diameter. This resulted in an artificial virus-like particle vaccine suspension, which was subsequently tested in vitro (hemagglutination assay) and in vivo in a mouse model [14].

The highly pathogenic avian influenza (A/H5N1) virus is emerging as a leading candidate for a worldwide disease outbreak despite of its speedy proliferation through avian hosts and the ability to directly transmit infection to humans [15]. Haemagglutinin (HA) is the major surface protein of the A/H5N1 virus. HA contains virus-neutralizing epitopes and, therefore, it is the main antigen for vaccine development against A/H5N1 [16]. Generation of a universal vaccine that induces broad immunity response and broad protective against several A/H5N1 virus strains from different clades is attracting goal for researchers. In a previous study carried out by Thi Pham and colleagues (2019), a novel artificial HA sequence (H5.c2) generated by a method, namely, computationally optimized broadly reactive antigen (COBRA) H5.c2 sequence was selected as a representative of the subclades 2.3.2.1, 2.3.2.1a, 2.3.2.1b, and 2.3.2.1c of A/H5N1 strains from 2005 to 2015 in Vietnam. The H5.c2 protein was successfully expressed in *Nicotiana benthamiana* via agroinfiltration [17]. However, the immunogenicity of the H5.c2 protein was not evaluated due to its low expression level.

To follow up on our previous initial work, in the current study, the effects of size and surface properties of NDs on the immunogenicity of the H5.c2 protein in mice were assessed. The plant based-purified H5.c2 protein was conjugated with different ratios of NDs in different sizes and surface properties. The optimized H5.c2:NDs were selected in vitro by hemagglutination assay, Sodium dodecyl sulfate-polyacrylamide gel electrophoresis (SDS-PAGE), Western blot analysis, and the changes in size and zeta-potential of the particles before and after protein coating. The optimal mixture of H5.c2:NDs was then validated in vivo in mice. Finally, the H5.c2-specific-antibody and neutralizing antibody responses elicited in mice vaccinated with the optimal H5.c2:NDs conjugates, free H5.c2 and NDs were evaluated by ELISA, Western blot, and hemagglutination inhibition assay.

## 2. Materials and Methods

### 2.1. Nanodiamond Preparation

Production of surface oxidized nanodiamonds (ND40 and ND100): The crude nanodiamond purchased from FNDbiotech (Taiwan) was first surface-functionalized with oxygen containing groups under microwave heating (~100 °C) in a mixture of H_2_SO_4_ and HNO_3_ with the volume ratio of 3:1 (~100 °C). This harsh treatment was based on the protocol reported in a former publication [4]. A microwave reactor (100 W, Model Discover, CEM, Cridersville, OH, USA) was used to heat the material for 2 h. Subsequently, the remaining acids were diluted carefully and the NDs then were collected. The microwave reactor was put in a chemical fume hood to shield the operator from NO_2_ contamination during working process. Next, NaOH 1N was added into the mixture. ND was separated from the mixture by 15,000× *g* centrifugation for 2 h. NDs were then resuspended with deionized water (DI-H_2_O). The ND100 fraction was collected by centrifugation of the suspension at 10,000× *g* for 5 min, and the remaining suspension was centrifuged at 15,000× *g* for 2 h to collect ND40. The obtained NDs are washed with DI-H_2_O twice and resuspended again in DI-H_2_O for use.

Preparation of ND4: The aqueous DND colloids (NanoAmando) were provided from New Metals and Chemicals (Tokyo, Japan). NanoAmando was used as obtained, containing scattered DND particles dissolved in water (size: 3.7 nm in diameter, concentration: 5% DND) after optimization of the deagglomeration. The ND4 was characterized with positive surface charge and Zeta potential of +37 mV [9].

### 2.2. Production of Recombinant the H5.c2 Antigen

In the previous study, Thi Pham and colleagues (2019) designed a novel artificial H5 sequence based on selection for the most common, conserved, consensus, or recent epitope after comparing all of the HA sequences of A/H5N1 subclade 2.3.2.1, 2.3.2.1a, 2.3.2.1b, and 2.3.2.1c recorded in Vietnamese poultry farm from 2005 to 2015 [17]. The amino acid sequence of H5.c2 protein was provided in the Supplementary 1. Recombinant H5.c2 protein fused with GCN4pII at its C-terminator was expressed transiently in *N. benthamiana*, purified via immobilized affinity chromatography (IMAC) and size exclusion chromatography (SEC) according to the previous protocols described by Thi Pham and colleagues [17]. The purity of the H5.c2 protein after purification was over 95%, as shown by SDS-PAGE (Supplementary 2). The purified H5.c2 protein was stored at –20 °C before use. The physical characterization of H5.c2 protein was predicted by using the Bachem’s peptide calculator (https://www.bachem.com/knowledge-center/peptide-calculator/, accessed on 20 May 2021).

### 2.3. Conjugation of H5.c2 Antigen onto the Surface of Nanodiamonds

H5.c2:ND conjugates were synthesized by the physical adsorption method. Various H5.c2:ND conjugate (*w*/*w*) of 1:1, 1:3, 1:6, 1:12, 1:24, and 1:48 were diluted in Phosphate Buffer Saline buffer (137 mM NaCl, 2.7 mM KCl, 10 mM Na_2_HPO_4_, 1.8 mM KH_2_PO_4_, pH 7.4, PBS). During coating, sonication was applied for 30 min. Centrifugation at 13,000× *g* for 5 min was used to collect the H5.c2:NDs conjugates. The non-binding complexes were then removed by washing twice in PBS buffer.

### 2.4. H5.c2:ND Conjugate Characterization

#### 2.4.1. Hemagglutination Assay

Hemagglutination assays were carried out to evaluate the ability to agglutinate chicken red blood cells (RBC) of HA antigen. The hemagglutination assays were carried out according to the guideline of the World Organization for Animal Health [18]. Briefly, all wells of a plastic V-bottom microtiter plate were introduced with 50 µL of PBS. Next, in the first well of plate, antigens (50 µL) were placed. Across the entire rows, a total 50 µL of two-fold serial dilution was performed. Following the introducing with 50 µL of 1% RBCs into each well of the microtiter plate, the incubation at 25 °C was conducted. After 30 min of incubation, the results were visualized. One hemagglutination unit was described as the endpoint dilution that results in complete hemagglutination (HAU).

#### 2.4.2. Size and Zeta-Potential Measurements

To evaluation the change in size and Zeta potentials of NDs before and after coating with H5.c2 protein, a nanoparticle size analyzer (Delsa@NanoC, Beckman Coulter Inc., Fullerton, CA, USA) was utilized. Before measurement process, NDs and H5.c2:NDs were completely diluted to the concentration of 100 µg/mL in DI-H_2_O.

#### 2.4.3. SDS-PAGE and Western Blot of H5.c2-NDs

The H5.c2 protein (100 ng/µL) was conjugated with ND4, ND40, and ND100 at different ratios (1:12, 1:3, 1:12, *w*/*w*), respectively. To remove non-binding products, the resulting mixtures were washed twice in PBS. The same volume of the H5.c2 protein (1.5 µg) and H5.c2:ND conjugates were diluted in SDS-sample buffer 1X (3% SDS, 1.5% DTT, 0.1% bromophenol blue, 0.5 M Tris-HCl pH 6.8, 10% glycerol), then separated by 4–10% SDS-PAGE gel. Casting and running of protein gels were performed via BioRad’s Miniprotean II (Laemmli systerm). Proteins on the gel were stained by Coomassie brilliant blue G-250 (Bio Basic Inc., Markham, ON, Canada).

For detection of protein by Western blots, proteins were transferred to PVDF membrane (Thermo Scientific). Proteins were then executed via the procedure reported by Gahrtz and Conrad [19]. Briefly, the membrane was blocked with a PBS buffer containing 5% (*w*/*v*) fat-free milk for 2 h at room temperature. Next, the membrane was incubated for 2 h in PBS buffer including 5% (*w*/*v*) fat-free milk and monoclonal anti-c-myc antibody that was diluted 1:50 times. The membrane was washed three times with PBS buffer before incubation for 1 h at room temperature with the goat anti-mouse IgG secondary conjugated HRP at the dilution of 1:2000 times in 5% (*w*/*v*) fat-free milk dissolved in PBS buffer. Signals were visualized by incubation of membrane for 15 min in the dark with 3,3-diaminobenzidine (DAB, Thermo Scientific, Rockford, IL, USA) that was dissolved in 0.05 M Tris–HCl and 0.04% hydrogen peroxide. The intensity of H5.c2 band signals were analyzed by ImageJ software.

### 2.5. Mouse Experiment

The 6–8-week-old female BALB/C mice (five per group) were vaccinated with 2.5 µg of purified H5.c2 (group 1), a combination of 2.5 µg of purified H5.c2 and 7.5 µg of ND40 [1:3 (*w*/*w*), group 2], and another mixture of 2.5 µg of purified H5.c2 and 30 µg of ND100 [1:12 (*w*/*w*), group 3] on a schedule of 1, 14, 28 days via subcutaneous route. The mice of the negative control group were immunized with a mixture of PBS and 30 µg of NDs (group 4). In addition, to evaluate the effect of ND4 on the immunogenicity of H5.c2, a mixture of 2.5 µg of purified H5.c2 and 30 µg of ND4 [1:12 (*w*/*w*), group 5] was used for immunization in mice. In the first immunization, complete Freund’s adjuvant was used for the formulation of antigens. The incomplete Freund’s adjuvant was mixed with antigens in the second and the third immunizations. Seven days after the booster vaccination, mouse sera were obtained for ELISA tests, Western blot and hemagglutination inhibition assay.

### 2.6. ELISA

To evaluate H5.c2-specific-IgG antibody responses in mouse sera, 100 µL of 100 ng H5.c2 (purified by SEC) was applied in microtiter plates (ImmunoPlate Maxisorp, Nalgene Nunc International, Roskilde, Denmark), and the plates were incubated overnight at 4 °C. Next, the plates were blocked with PBS buffer containing 3% (*w*/*v*) BSA, 0.05% (*v*/*v*) Tween20 for 2 h. The plates were then added with 100 µL of PBS buffer including 1% (*w*/*v*) BSA, 0.05% (*v*/*v*) Tween 20 and the mouse sera with the dilution of 1:16,000, followed by plate incubations for 2 h at 25 °C. Each mouse serum was applied 3 times in the plate. A goat anti-mouse IgG conjugated horseradish peroxidase (HRP) diluted 2000 times in PBS buffer containing 1% (*w*/*v*) BSA, 0.05% (*v*/*v*) Tween 20 was applied in the plates for 1 h at 25 °C after washing the plates with PBS buffer containing 0.05% (*v*/*v*) Tween 20. The signals were detected by adding the 1-Step™ Ultra TMB-ELISA Substrate Solution (Thermo Fisher Scientific, Vilnius, Lithuania). The plates were then incubated for 20 min before the addition of 1 M HCl to stop reaction. The microplate reader (Biorad Laboratories Inc., Hercules, CA, USA) was used for measurement of the absorbance signal at 450 nm. The values of all mouse sera in the ELISA plates were normalized after subtracting the background values for BSA.

### 2.7. Detection of H5.c2-Specific-IgG Antibody Responses by Western Blot

To detect the H5.c2-specific-IgG mouse antibodies, each 500 ng of H5.c2 protein (purified by SEC) was applied on a 4–10% SDS-PAGE gel. A semidry Quick Blotter was used to switch proteins to a PVDF membrane (Thermo Scientific, Rockford, IL, USA). The membrane was blocked with PBS buffer containing 5% (*w*/*v*) fat-free milk for 2 h at room temperature. The membrane was cut into five lanes that were then incubated with mouse serum mixture of each group at the dilution of 1:500 for 2 h. The membrane was washed three times for 15 min before incubation with goat anti-mouse IgG conjugated HRP (Thermo Fisher Scientific, Rockford, IL, USA) at the dilution of 1:2000 for 1 h. Before detection of specific signals, the membrane was washed three times for 15 min, then were incubated in the dark for 10 min with 0.05 M Tris-HCl buffer containing DAB (Thermo Scientific, Rockford, IL, USA) and 0.04% H_2_O_2_.

### 2.8. Hemagglutination Inhibition Assay

The hemagglutination inhibition (HI) assay was similarly performed using a standard procedure [18]. The inactivated virus strain (IBT-RG02) was used for the HI assay. The IBT-RG02 strain was produced by reverse genetic technology using HA and neuraminidase (NA) proteins of the HPAI H5N1 (A/duck/Vietnam/HT2/2014(H5N1) of the clade 2.3.2.1c in the period 2012–2014 in Vietnam [20]. The HA amino acid sequence similarities between the synthetic H5.c2 and native H5 of A/duck/Vietnam/HT2/2014(H5N1) strains is 99.4%. Firstly, all wells of a plastic V-bottom microtiter plate were placed 25 µL of PBS. Next, in the first well of plate, antigens (25 µL) were placed. The eight-well rows were then subjected to two-fold serial dilutions. A 25 µL of the last dilution was discarded. The 4 HAU of the inactivated IBT-RG02 strain (25 µL) was subsequently placed in each well. The reaction was incubated for 30 min at 25 °C. Finally, each well received 25 µL of 1% RBCs. The results were read after incubating for 30 min at 25 °C. The reciprocal of the highest dilution of serum that could fully inhibit hemagglutination was identified as the HI titer.

### 2.9. Statistical Analysis

The ELISA’s results were statistically analyzed by *t*-test of the Sigma Plot software (Chicago, IL, USA). The X ± standard deviation (SD) was shown for the mean difference of the sample’s data. The statistical differences were identified if *p* values < 0.05.

## 3. Results

### 3.1. Production and Biological Characterization of the H5.c2:ND Conjugates

Since NDs can conjugate to various proteins or antigens but not selective conjugation, it is a requirement to purify proteins or antigens before mixing with NDs. In this study, H5.c2 protein was expressed in *N. benthamiana*, then purified by IMAC and SEC. The high purity of H5.c2 protein was validated by SDS-PAGE (Supplementary 2). H5.c2 protein was characterized as a trimeric protein containing 5 glycosylation sites (data not shown). The appearance of H5.c2 bands obtained in the SDS-PAGE was higher than its theory that was predicted with monomer’s molecular weight of 64 kDa. It can be explained by the fact that glycosylation sites can affect the movement of the protein during electrophoresis.

To evaluate biological activity and select the optimal mixture of the H5.c2:ND complexes, hemagglutination assays were carried out. In general, the hemagglutination assay revealed the stronger ability to agglutinate chicken RBCs of H5.c2:ND conjugates (except H5.c2:ND4) at ratios from 1:1 to 1:48 (*w*/*w*) in comparison to free H5.c2 (Table 1).

**Figure 1:**
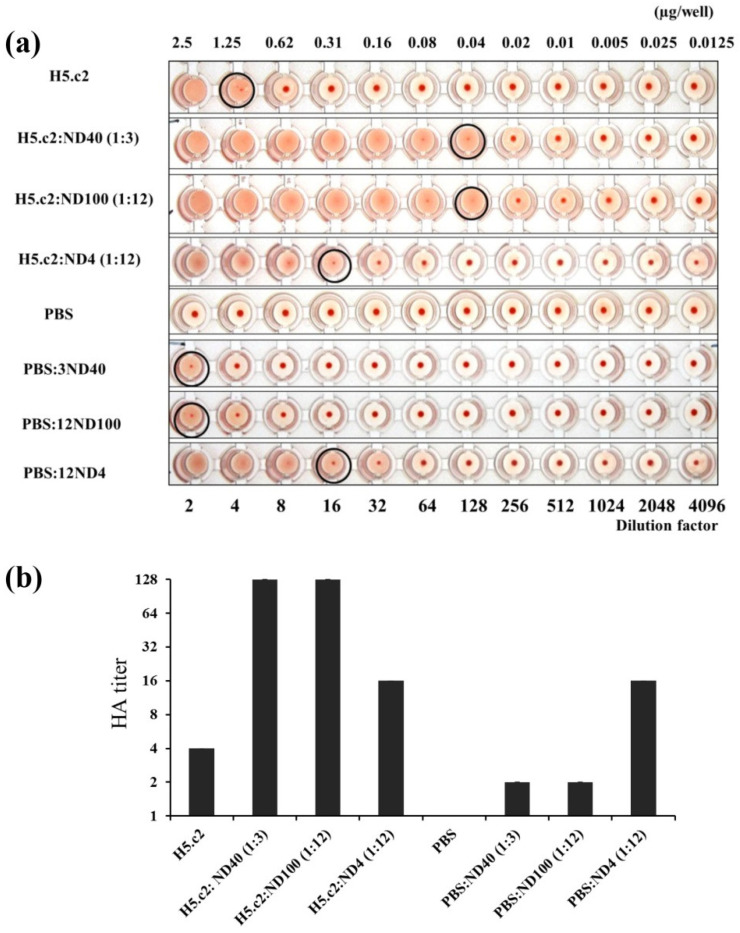
Bio-functional characterization of free H5.c2 protein and the mixture of H5.c2 and NDs via a hemagglutination assay. (**a**) 50 µL of the H5.c2 protein (50 ng/µL) or the H5.c2:ND4, H5.c2:ND40, H5.c2:ND100 conjugates were used for the hemagglutination assay. HAU of each sample, were presented by black circle symbols. (**b**) The hemagglutination assay was carried out with three replications for each sample. The columns presented the average HA titer value of the samples and SD.

Of these, the mixture of H5.c2:ND40 and H5.c2:ND100 at the ratios of 1:3 and 1:12 (*w*/*w*), respectively, expressed the highest HAU of 64 after subtract the HAU of the PBS:ND, whereas the free H5.c2 protein agglutinated RBCs with HAU of 4 (Figure 1).

The HAU of these H5.c2:ND complexes were both 16 times higher than the free H5.c2 protein. In contrast, there was no increase in HA titer of the H5.c2:ND4 mixture when the amount of ND4 at H5.c2:ND4 ratios were increased from 1:1 to 1:12 (*w*/*w*), or even 1:48 after subtracting the HAU of PBS:ND4. The successful collection of H5.c2:N4 mixture using centrifugation was confirmed (Supplementary 3). The PBS:NDs at the ratios of 1:3 to 1:48 (*w*/*w*) agglutinated chicken RBCs at HA titer from 2 to 16, especially PBS:ND4 at the ratios of 1:12 to 1:48 (*w*/*w*) agglutinated chicken RBCs at HA titer of 16. These results reveal that ND40, ND100, and ND4 at a minimum amount of 7.5 µg, can interfere in the hemagglutination assay resulting in pseudo agglutination with RBC in the case without the presence of HA antigen. These results also indicate that ND4 particles do not have any impact in enhancing HA titer of the H5.c2 protein. In contrast, ND40 and ND100 have effects in improving the bio-functional of the H5.c2 protein, and the optimal H5.c2:ND ratios are 1:3 (*w*/*w*) and 1:12 (*w*/*w*) for the H5.c2:ND40 and H5.c2:ND100 conjugates, respectively.

**Figure 2:**
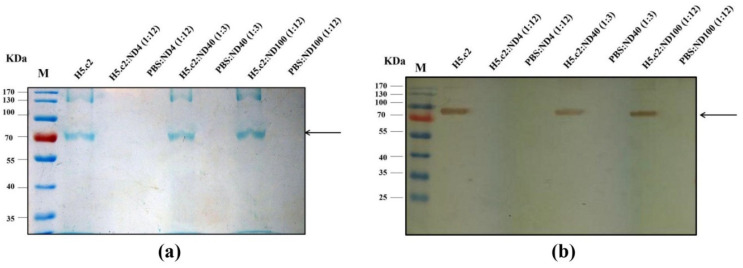
Characterization of H5.c2:ND conjugates at different ratios by SDS-PAGE and Western blot. (**a**) The same volume of the H5.c2 protein (1.5 µg) and H5.c2:ND conjugates separated by 4–10% SDS-PAGE and stained by Coomassie brilliant blue G-250; (**b**) The H5.c2 protein and H5.c2:NDs conjugates were then transferred to the PVDF membrane, and detected by using the anti-c-myc monoclonal antibody followed by goat anti-mouse IgG HRP.

Proof that the H5.c2 protein could bind onto the surface of ND4, ND40, and ND100 after protein coating, the final optimized mixtures of H5.c2:ND4, H5.c2:ND40, and H5.c2:ND100 conjugates (after washing with buffer to remove non-binding protein) were demonstrated by SDS-PAGE and Western blot (Figure 2).

As assumed, the results confirmed that H5.c2 protein did not coat onto the surface of ND4. In contrast, the strong binding potential of the H5.c2 protein on the surface of ND40 and ND100 at ratios of 1:3 and 1:12, respectively, was detected at over 80% compared to the free H5.c2 protein. These results confirmed again the high binding of negatively charged particles (ND40 and ND100), but not positively charged particle (ND4) with the H5.c2 protein at the same condition.

### 3.2. Size and Surface Properties of the H5.c2: ND Conjugates

**Figure 3:**
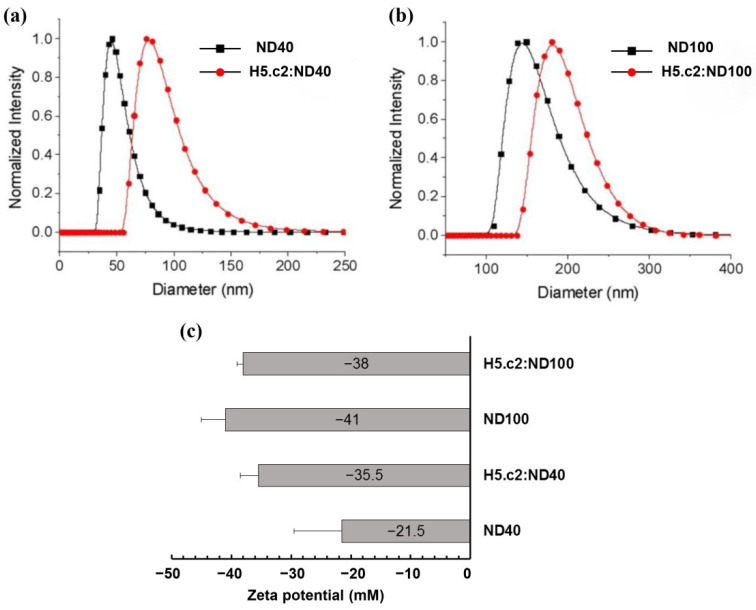
Size and surface properties of H5.c2 and H5.c2-ND conjugates. (**a**,**b**). The size distribution of the re-suspension of ND40 and ND100 before and after coating with the H5.c2 protein in DI-H_2_O was measured by particle analyzer (Beckman Coulter); (**c**) Zeta potential of NDs and H5.c2:ND conjugates.

H5.c2 protein was predicted with an isoelectric point of 6.3 and a net charge of −5.2 at pH 7.0 according to Bachem’s peptide calculator (https://www.bachem.com/knowledge-center/peptide-calculator/, accessed on 20 May 2021). The binding between the H5.c2 protein and NDs was also evaluated by the differences in the size and zeta-potential before and after coating with the antigen protein (Figure 3). For the particle size measurements, the H5.c2:ND conjugates were dissolved in DI-H_2_O.

The results in Figure 3a,b showed that ND40 and ND100 have size ~40–70 nm and ~100–250 nm in diameter, respectively. The sizes of ND40 and ND100 increase approximately 40 nm in diameter after coating with H5.c2 protein. It means that the H5.c2 protein was coated significantly onto the surface of ND40 and ND100 particles. The effect of the H5.c2 protein coating on the surface chemistry of NDs was investigated further using Zeta-potential measurements. The results in Figure 3c indicated that ND40 and ND100 had negatively charged surfaces. Zeta-potentials of ND40 and ND100 were ~−21.5mV and ~−41 mV, respectively. When coating with the H5.c2 protein, the H5.c2:ND complexes still had negative Zeta-potential, and more interestingly the surface charge of the two complexes H5.c2:ND40 and H5.c2:ND100 was not significantly different since Zeta potentials of the H5.c2:ND40 and H5.c2:ND100 conjugates were~−35.5mV and ~−38 mV, respectively.

### 3.3. H5.c2:ND40 and H5.c2:ND100 Mixtures Stimulated Stronger H5.c2-Specific-IgG Antibody Responses in Comparison to the Free H5.c2 Protein

**Figure 4:**
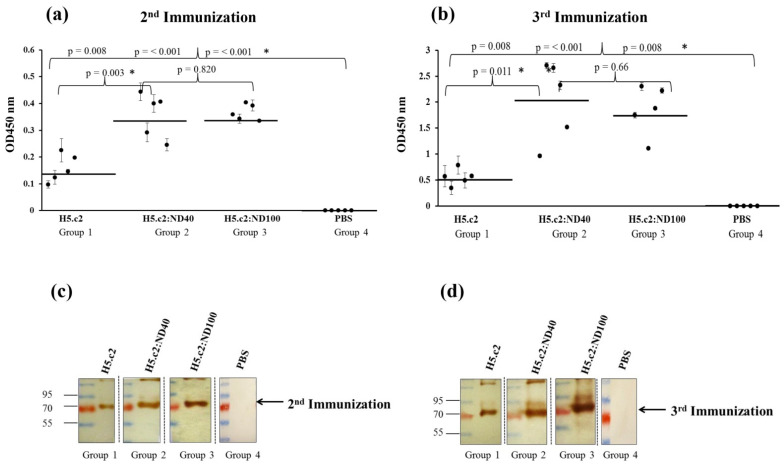
Determination of the H5.c2-specific-IgG antibody responses in mice (*n* = 5 per group) via ELISA (**a**,**b**) and Western blot (**c**,**d**). The *t*-test (SigmaPlot) was used for statistical analyses in ELISA. A single dot showed the mean ELISA value of a single mouse serum with three replications. The mean ELISA values of the tested groups were presented as the bars. The statistically significant differences with *p* < 0.05 were indicated by * symbol.

H5.c2-specific-IgG antibody responses in mice vaccinated with free H5 (group 1), H5.c2:ND40 conjugate (1:3, (*w*/*w*), group 2), H5.c2: ND100 conjugate (1:12, (*w*/*w*), group 3), as well as PBS:ND (group 4) were evaluated via ELISA and Western blot (Figure 4).

The ELISA results indicated that a stronger H5.c2-specific-IgG response was induced in mice vaccinated with the H5.c2:ND40 (group 2) and H5:ND100 (group 3) conjugates compared to the free H5.c2 protein (group 1) with *p* < 0.05 (Figure 4a,b). Notably, ELISA results reveal that after the second immunization, H5.c2-specific-IgG antibody responses in mice immunized with the H5.c2:ND40 and H5.c2:ND100 conjugates were over 2 times stronger than that with the free H5.c2 protein. Moreover, after the third immunization, the H5.c2:ND40 and H5.c2:ND100 conjugates induced H5c.2-specific-IgG antibody responses that were three times stronger than those of the free H5.c2 protein. However, there was no significant difference in the H5c.2-specific-IgG antibody response vaccinated in mouse groups 2 and 3 (*p* = 0.820 and *p* = 0.66 > 0.05 after the second and the third immunization, respectively). As expected, there was no H5.c2-specific-IgG response stimulated in the negative control group mice injected with PBS:ND (group 4).

The Western blot results in Figure 4c,d confirmed that strong signals were obtained in mice vaccinated with the H5.c2:ND40 conjugates (group 2) and H5.c2:ND100 conjugates (group 3) compared to those in mice vaccinated with free H5.c2 protein (group 1). It means that H5.c2:ND40 and H5.c2:ND100 stimulated stronger H5.c2-specific-IgG antibodies than free H5.c2 protein. The H5.c2-specific antibody responses increased considerably after the third immunization. In contrast, there was no signal detected in the negative control group mice.

### 3.4. Stronger Neutralizing Antibody Responses Were Induced by the H5.c2:ND40 and H5.c2:ND100 Conjugates, as Opposed to the Free H5.c2 Protein

**Figure 5:**
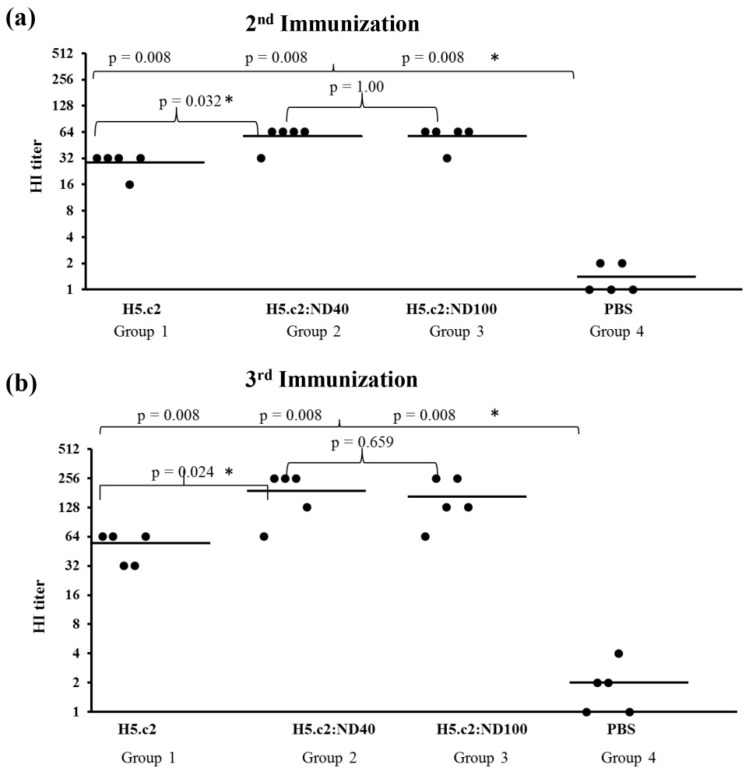
Determination of neutralizing antibody responses in mouse sera by hemagglutination inhibition assay. The inactivated virus strain (designated as IBT-RG02) produced by reverse genetic using HA and NA from the clade 2.3.2.1c (A/duck/Vietnam/HT2/2014(H5N1)) was used for HI assay. Serum from a single mouse after the 2nd immunization (**a**) and the 3rd immunization (**b**) was used. A single dot presented the mean HI titer of each mouse serum with three replications. The bars showed the HI geometric mean titers (HI GMTs) value of the tested groups. * indicated a statistically significant difference with *p* < 0.05.

To measure the neutralizing antibodies produced in mice as a result of vaccination with the H5.c2:ND40 (group 2), H5.c2:ND100 (group 3) conjugates and the free H5.c2 protein (group 1), we performed hemagglutination inhibition assays (Figure 5).

When the stronger neutralizing antibodies against the H5.c2 protein presented in mouse sera, the higher HI titer was obtained. As expected, after the second immunization, the neutralizing antibody responses were statistically significant, being two times stronger in mouse groups 2 and 3 than in mouse group 1 (*p* < 0.05) (Figure 5a). The HI geometric mean titers (HI GMTs) of mouse groups 2 and 3 were equal to 57.6, while the HI GMTs of mouse group 1 had a low result of 28.8 after the second immunization. In addition, neutralizing antibody responses were enhanced in mouse vaccine groups 1, 2, and 3 after the third immunization. HI titers for mouse groups 2 and 3 vaccinated with the H5.c2:ND40 and H5.c2:ND100 conjugates, respectively, were 3–3.5 times higher than mouse group 1, immunized with the free H5.c2 protein after the third immunization (Figure 5b). The HI GMTs of mouse groups 2 and 3 were 192 and 166.4, respectively, while the HI GMTs of mouse group 1 were only 54.9. In contrast, the neutralizing antibody responses induced in the negative mouse control group 4 were as low as 1.4 and 2.0, respectively, after the second and third immunization.

### 3.5. ND4 Does Not Have any Effect on the Immunogenicity of H5.c2 in Mice

The effect of ND4 on the immunogenicity of the H5c.2 protein was evaluated via ELISA, Western blot, and hemagglutination inhibition assay. These results showed that H5.c2 specific-antibody responses and neutralizing antibody responses in the mouse group 5 immunized with the mixture of H5.c2:ND4 were similar to those in the mouse group 1 injected with the free H5.c2 protein (Supplementary 4). Therefore, ND4 did not affect to the immunogenicity of the H5.c2 protein in mice.

## 4. Discussion

A/H5N1 virus not only poses a serious danger to avian hosts, but also has the potential to directly infect humans [15]. Due to viral antigenic drift, it is urgently needed to develop universal influenza vaccines that induce protective immunity for a long-term against distant influenza virus strains [21]. Previous reports showed that the polyvalent VLP vaccine based on HA COBRA that represented different clades of A/H5N1 elicited a strong immunity response against different clades of A/H5N1 [22,23]. However, production of a native VLP vaccine based on native HA is time-consuming and complex. Nanoparticle displaying antigen-forming synthetic VLP is considered to be an alternative approach to conventional vaccines, since it supports antigen stabilization. Previous studies showed that a synthetic-VLP formed by conjugating proteins with nanoparticles enhanced immune response [14,24]. H5.c1 appeared for the A/H5N1 subclades 1.1, 1.1.1, and 1.1.2 induced strong immune responses against different A/H5N1 strains that belong to distinct clades [17]. Based on previous results, in order to improve immune responses via enhanced antigen size, as in the case of synthetic-VLP formation, H5.c2 protein appeared for the HA protein of A/H5N1 subclades 2.3.2.1, 2.3.2.1.a, 2.3.2.1.b, 2.3.2.1.c was used to conjugate NDs of different surface properties (positive and negative) and sizes.

In the present study, the H5.c2:ND40 (1:3, *w*/*w*) and H5.c2:ND100 (1:12, *w*/*w*) conjugates agglutinated chicken RBCs at equal HA titers of 64, which are 16 times greater than that of free H5.c2 (Figure 1, Table 1). These data indicate a greater capacity for H5.c2 conjugation and the preservation of its antigenic properties and antigen bio-function with negatively charged ND particles. Our synthetic H5.c2:ND40 and H5.c2:ND100 exhibited the potential for agglutination RBCs at a minimum total protein amount of 0.04 μg which is 31.25 times lower than that of H5.c2 free protein of 1.25 μg. The differentiated HA titer is modulated mainly by charge and not size, raising questions about the mechanism of action in vitro experiments. Several reports have demonstrated that higher HA titers are obtained in lower HA protein amounts when the HA antigen size was increased via H5 oligomeric or H5-VLP formation [14,25,26]. Thus, H5.c2 antigen size increased after coating negatively charged ND40 and ND100.

Moreover, we provided supporting evidence for the notion that charged-specific interactions can indeed mediate H5.c2 protein adsorption onto ND’s surface. Our data confirmed that the mixture of H5.c2 was strongly conjugated on the surface of negatively charged ND40 and ND100 but not with positively charged ND4. These results were almost identical to a previous study of Lin and co-workers. When compare the attachment of proteins (myoglobin, bovine serum albumin, and insulin) to these two types of NDs (DNDs and HPHT-NDs), the results showed that the HPHT-NDs (negative charged NDs) had strong protein-surface interaction with all three proteins [9]. In contrast, DNDs had a tiny protein-surface interaction with the proteins [9]. In addition, several previous studies indicated that oxidized HPHT-NDs are negative charged, can bind non-covalently but strongly with proteins through hydrogen bonding, the combination of electrostatic force and hydrophobic interactions [12,14].

To test the immunogenicity of the H5.c2 protein conjugate with negatively charged NDs compared to the free H5.c2 protein, the optimized mixture of H5:ND40 and H5:ND100 were immunized in a mouse model (in vivo experiments). As expected, the mixture of H5.c2:ND40 and H5.c2:ND100 elicited levels of H5.c2-specific-IgG antibodies that were statistically significantly higher than those of the free H5.c2 protein (as shown in Western blot and ELISA results, Figure 4a,b) and statistically significant stronger neutralizing antibodies against A/H5N1 virus clade 2.3.2.1c (as shown by hemagglutination inhibition assay, Figure 5) which suggests a potential effect inherent to ND40 and ND100 (with negative charged surface). The elicitation of this strong and specific immune response might be explained by several reasons. A previous study showed that the HPHT-NDs with negatively charged NDs had significant surface associations with proteins and created a thin layer on surface of the particles [9]. The synthetic HA-VLPs formation or particles enhances HA-specific-IgG immune responses by improving the presentation of HA antigen to the immune system which are then delivered to draining lymph nodes [14]. Moreover, Garcia-Bennett and colleagues reported that NDs can interact with proteins in a biological milieu, forming protein corona by electrostatic attractions between proteins and NDs [27]. In addition, VLPs can act as adjuvants by improving antigen processing pathways [28]. Although the H5.c2-specific-IgG antibodies and neutralizing antibodies induced in the mouse group immunized with the H5.c2:ND40 conjugate was higher than that of the H5.c2:ND100 conjugate, there was no significant difference between the two groups. It can be explained by the fact that there was no difference in the physical characteristics of ND40 and ND100 after conjugating with the H5.c2 protein.

These results indicate the good effects of oxidative NDs as antigen carriers for H5.c2 antigen. NDs are used instead of other nanomaterials because of some unique and notable benefits. Firstly, antigens can be conjugated onto the surface of NDs very fast and economically. The formation of antigen-NDs conjugates is completed in less than 10 min and no chemical reactions are needed to link antigens to NDs. The conjugate formation process through the physical adsorption method is a green chemistry technology. Many other substances (e.g., magnetic nanomaterials, gold nanoparticles, polymeric nanoparticles) require chemical reactions or labor-intensive to carry protein antigens [14]. Secondly, the oxidative NDs have a unique surface that has been characterized to have both hydrophobic area and area of functional oxygen groups (–COOH, –CO, –OH) [4,29]. Thanks to the ND unique surface chemistry, our NDs can conjugate many different types of antigen proteins, both soluble proteins, and micelle-solubilized membrane proteins [4,13]. Earlier studies also showed that proteins but not short peptides bind strongly to the surface of oxidative NDs [4,13,30]. Almost proteins (MW > 10 KDa) do not dissociate from NDs under non-destructive conditions. In contrast, short peptides (e.g., tryptic peptide digest) are easily detached from NDs surface in pH 7.0 or basic pH. This may also explain why H5.c2:NDs are stable and effectively processed by the immune system to boost the immune response in comparison with free H5.c2 antigen. Thirdly, NDs can be produced to be fluorescent with the many [N-V] centers inside its lattice, or fluorescent ND (FND). A previous publication demonstrated the usage of FND conjugated with A27 vaccinia surface antigen to study the interaction of the viral recombinant with cultured mammalian cells to mimic virus host cells [30]. As [N-V] centers of FNDs have unique photochemical properties such as no photo-bleaching, no photo-blinking, highly bright and inert to the environment, and thus FNDs can be used to track the antigen trafficking both in vitro and in vivo. Consequently, further study will perform to use FNDs for evaluation of the interaction of H5.c2:NDs with the immune system and the excretion of NDs from the body of animals.

Several publications reported about the excretion of NDs from our body after use. Rats were safe after vaccination with a single high dose of FDNP-(NV)-Z~800 (60 mg/kg) that were principally distributed in the liver, spleen, and the clearance (50%) happened after 33 min following the end of particle infusion [31]. Furthermore, another publication indicated that amino-functionalized NDs labeled with F-18 were excreted into the urinary tract after accumulation mainly in the lung, spleen, and liver [32]. In addition, the accumulation of FND was non-toxic even when a large quantity, up to 75 mg/kg body weight which is a far-excessive amount of NDs needed to prepare vaccines [33]. Taken together, the excretion of the NDs from the animal’s body is dependent on size, surface modification, and injection routes. Most previous studies indicate that NDs are safe and very biocompatible, especially oxidative NDs.

## 5. Conclusions

In summary, effects of the size and surface properties of NDs were successfully assessed by simply mixing recombinant H5.c2 protein with NDs in an optimized ratio. Our results demonstrate that the H5.c2 protein conjugates on the surface of negatively charged ND particles (ND40 and ND100), but not positively charged ND particles (ND4). The H5.c2:ND40 and H5.c2:ND100 conjugates induced stronger H5.c2-specific-IgG antibodies and neutralizing antibodies compared to the free H5.c2 protein. These results confirm the probable effects that negatively charged ND have improved immune response. Evaluating the size and surface properties of ND is necessary before the broad application of ND in nanovaccine development.

## Figures and Tables

**Table 1:** Bio-functional screening of H5.c2:ND conjugates by hemagglutination assay.

Complex Samples	H5.c2:ND Ratio	HAU	Complex Controls	ND Ratio	HAU	HAU of H5.c2:ND after Subtract HAU of PBS:ND
H5.c2	1:0	4	PBS	0	0	4
H5.c2:ND40	1:1	32	PBS:ND40	1:1	0	32
	**1:3**	**128**		**1:3**	**2**	**64**
	1:6	128		1:6	4	32
	1:12	128		1:12	4	32
	1:24	64		1:24	4	16
	1:48	8		1:48	8	0
H5.c2:ND100	1:1	4	PBS:ND100	1:1	0	4
	1:3	8		1:3	2	4
	1:6	32		1:6	2	16
	**1:12**	**128**		**1:12**	**2**	**64**
	1:24	64		1:24	4	16
	1:48	64		1:48	8	8
H5.c2:ND4	1:1	0	PBS:ND4	1:1	0	0
	1:3	2		1:3	2	0
	1:6	4		1:6	4	0
	1:12	16		1:12	16	0
	1:24	16		1:24	16	0
	1:48	16		1:48	16	0

## Data Availability

The datasets used and analyzed during the current study are available from the corresponding author on reasonable request.

## Supplementary Materials

The following are available online at: http://doi.org/10.5281/zenodo.4971389, Supplementary 1. H5.c2 amino acid sequence; Supplementary 2: Purification of H5.c2 from leaves of *N. benthamiana* by Immobilized affinity chromatography (IMAC); Supplementary 3: Collection H5.c2:ND4 conjugate by centrifugation at 13,000 rpm, 30 min; Supplementary 4: Determination of the effect of ND4 on the immunogenicity of H5.c2 protein in mice.
